# Acute Exercise-Induced Epinephrine Elevation Promotes Post-Learning Memory Consolidation: A Narrative Review of Mechanisms and Implementation Strategies

**DOI:** 10.3390/life16010013

**Published:** 2025-12-22

**Authors:** Yiwan Zhang, Xuewan Lin, Gen Li, Songtao Wang

**Affiliations:** School of Physical Education & Sports Science, South China Normal University, Guangzhou 510631, China; yiwanzhang1230@163.com (Y.Z.); 15816671136@163.com (X.L.)

**Keywords:** epinephrine, memory consolidation, exercise-induced, time window, cognitive enhancement

## Abstract

Memory function is susceptible to decline with age, stress, and neurological diseases, highlighting the importance of exploring effective and sustainable strategies to enhance memory consolidation. Epinephrine plays a key role in memory consolidation; acute, moderate elevations enhance memory, while chronic high levels are inhibitory. Given the limitations of pharmacological interventions, this study aims to investigate exercise as a non-pharmacological means to promote post-learning memory consolidation by inducing acute epinephrine release, focusing on its mechanisms and optimized implementation strategies. This narrative review systematically reviews evidence from neurophysiology, molecular biology, and behavioral experiments and finds that exercise can safely and controllably activate the sympathetic–adrenal system, leading to a rapid rise in epinephrine. The release kinetics align highly with the critical time window for memory consolidation. Moderate-intensity aerobic exercise implemented within 30 min post-learning can significantly improve memory retention. The mechanisms involve not only epinephrine enhancing synaptic plasticity and LTP by activating hippocampal β-adrenergic receptors, but also synergistic effects across multiple systems, such as promoting osteocalcin signaling, upregulating BDNF expression, inducing neurogenesis, and optimizing cerebral metabolism and blood flow. Evidence suggests that exercise, as a non-pharmacological intervention, significantly enhances post-learning memory consolidation through the precise modulation of epinephrine release and multi-system synergy, offering both high efficacy and safety. Future research should focus on developing precise exercise prescriptions based on individual characteristics and leveraging wearable devices and digital technologies to improve intervention adherence and applicability, promoting its widespread use in educational and clinical settings.

## 1. Introduction

Learning and memory are the cornerstones of human cognitive function, crucial for individual adaptation to the environment, knowledge acquisition, and social development [1]. However, memory function is prone to decline with age, cumulative stress, or neurological diseases, which not only severely impacts quality of life but also imposes a significant socio-economic burden. Consequently, exploring effective and sustainable strategies to enhance the memory consolidation process has become an important research direction in neuroscience and psychology.

Memory formation is a highly dynamic process involving multiple stages such as encoding, consolidation, and retrieval. The consolidation stage is particularly critical, as it transforms temporarily stored new memories into a stable, long-term form [2]. This process strongly depends on a specific neurochemical environment. In recent years, substantial evidence has indicated that neurotransmitters and hormones, particularly epinephrine, play a central role in regulating memory consolidation [3]. Epinephrine enhances synaptic plasticity and Long-Term Potentiation (LTP) by activating beta-adrenergic receptors in the hippocampus, thereby effectively strengthening memory traces [4]. However, its effect is characteristically a “double-edged sword”: an acute, moderate elevation can significantly enhance memory, whereas a chronic high level can inhibit hippocampal function and impair memory [5]. This highlights that precise regulation of the timing and magnitude of epinephrine release is crucial for maximizing cognitive benefits.

Given that pharmacological interventions may have side effects, dependency risks, and difficulties in precise timing control, non-pharmacological interventions are increasingly garnering attention [6]. Among various strategies, physical exercise demonstrates unique value. Exercise not only acts as a safe physiological stressor, inducing acute, controllable epinephrine release that perfectly matches the “time window” for memory consolidation [7], but its benefits extend far beyond. Research shows that exercise can also exert broad and profound effects on the brain through multi-system, multi-pathway synergistic actions, such as promoting hippocampal neurogenesis, enhancing Brain-Derived Neurotrophic Factor (BDNF) expression, and optimizing cardiovascular health and systemic metabolic state [8,9]. This dual advantage of simultaneously achieving an “acute epinephrine surge” and “long-term neural benefits” through a single behavioral intervention makes exercise a highly attractive “broad-spectrum” cognitive enhancement strategy.

Although existing research theoretically and mechanistically confirms the promoting effect of exercise on learning and memory, there are still shortcomings in translational application. Current studies mostly focus on verifying the overall effects of exercise, while the understanding of how to optimize key parameters of exercise intervention, such as type, intensity, dose, and timing to maximize its benefits, remains insufficient. Different exercise protocols, for example, Moderate-Intensity Continuous Training versus High-Intensity Interval Training, may significantly influence the kinetics of epinephrine release and subsequent neural benefits [10]. Furthermore, individual factors, including age, health status, and genotype, may also modulate the effects of exercise, indicating the necessity of developing personalized “exercise prescriptions” [11]. Therefore, quantifying the dose–response relationship between exercise intervention characteristics and memory improvement outcomes is essential for translating theoretical findings into actionable and efficient application strategies.

This article presents a narrative review that synthesizes existing evidence by which exercise promotes post-learning memory consolidation through inducing acute epinephrine release. Our literature selection focused on key databases, including PubMed and Web of Science, prioritizing human and animal studies published between 2000 and 2024 that investigated exercise-induced epinephrine release and its effects on memory consolidation. It will focus on two core questions: “Why is exercise an ideal intervention?” and “How can exercise protocols be optimized?” We will delve into the dual advantages of exercise in achieving an “efficient epinephrine surge” and providing “synergistic neural benefits.” Finally, moving from theory to practice, we will assess its application prospects and challenges in different scenarios, aiming to provide a solid theoretical framework and practical guidance for future research and for developing evidence-based, personalized cognitive enhancement programs.

## 2. Neurochemical Actions of Epinephrine: The Molecular Catalyst of Memory Consolidation

Epinephrine, as a key neuromodulator and stress hormone, plays a vital role as a “molecular catalyst” in the processes of memory formation and consolidation. Its effects exhibit significant concentration and time dependence. Moderate acute release strengthens memory by activating hippocampal β-adrenergic receptors and enhancing synaptic plasticity; conversely, chronic elevation impairs hippocampal function by inducing a persistent stress state. Therefore, its regulation of memory presents a typical “double-edged sword” characteristic, with the final outcome depending on whether its action mode is “acute” or “chronic.”

### 2.1. Activation of β-Adrenergic Receptors and Synaptic Plasticity

Epinephrine significantly enhances synaptic plasticity and LTP, thereby strengthening the processes of memory encoding and consolidation at the molecular and cellular levels by activating β-adrenergic receptors in the hippocampus. LTP, a phenomenon of sustained increase in synaptic strength, is widely considered the neural basis of learning and memory. Upon binding of epinephrine to β-adrenergic receptors, adenylate cyclase is activated, increasing intracellular cyclic adenosine monophosphate (cAMP) levels, which in turn activate the protein kinase A (PKA) signaling pathway. This signaling cascade ultimately leads to the phosphorylation of the transcription factor cAMP-Response Element Binding Protein, regulating the expression of a series of genes related to synaptic plasticity and memory [12]. Studies show that peripheral administration of epinephrine or direct infusion of β-receptor agonists, such as isoproterenol, into the hippocampus can enhance memory retention, and β-receptor antagonists can block these effects, further validating the central role of β-receptors in memory modulation [13]. Furthermore, epinephrine enhances. N-methyl-D-aspartic acid receptors function by modulating glutamatergic neurotransmission, promoting calcium ion influx, thereby inducing the generation and maintenance of LTP [14]. It is noteworthy that the effects of epinephrine on different subregions of the hippocampus vary, with the most significant impact on synaptic plasticity observed in the dentate gyrus and CA3 region, areas closely associated with contextual and spatial memory formation [15]. Therefore, by precisely regulating synaptic plasticity and LTP processes within the hippocampus, epinephrine acts as an indispensable “molecular catalyst” in memory consolidation.

### 2.2. The Double-Edged Sword Effect of Stress on Memory Formation

**Figure 1:**
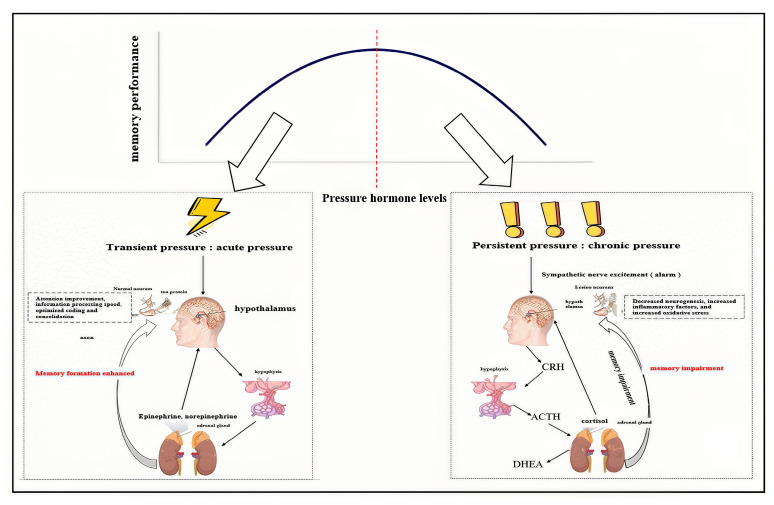
Different effects of acute and chronic stress on memory function: Double-edged sword effect. This figure compares the opposite effects of acute stress (left) and chronic stress (right) on hippocampal function and memory through different neuroendocrine mechanisms. Acute stress optimizes memory consolidation by rapidly releasing catecholamines, while chronic stress impairs hippocampal structure and function by sustaining high levels of cortisol. Original schematic summarizing the contrasting effects of acute vs. chronic stress on memory, inspired by models from McGaugh and Roozendaal (2002) [13] and McEwen (2007) [20].

Moderate acute elevation of epinephrine induces a state of “neurochemical acute stress.” This transient stress response significantly enhances memory formation by increasing attention, alertness, and information processing efficiency. However, chronic or excessive stress exerts inhibitory effects on the hippocampus through sustained high levels of cortisol and epinephrine, highlighting the critical importance of “acute” and “moderate” characteristics in stress-related memory modulation. Under acute stress, the rapid release of epinephrine and noradrenaline activates the locus coeruleus–noradrenergic system, enhancing neural activity in the prefrontal cortex and hippocampus, and optimizing the encoding and consolidation of information related to environmental threats [16]. This effect conforms to the classic “inverted U-shaped” dose–response curve, where moderate levels of stress hormones enhance memory, while very high or very low levels impair it. From an evolutionary perspective, the acute stress response prioritizes the processing of survival-relevant information, thereby promoting adaptive behavior [17]. In contrast, chronic stress leads to persistently elevated glucocorticoids, such as cortisol, causing dendritic atrophy of hippocampal neurons, reduction in synaptic connections, and inhibition of neurogenesis, ultimately impairing memory function and cognitive flexibility [18]. Studies also indicate that chronic stress exacerbates neurodegenerative changes in the hippocampus by promoting the release of pro-inflammatory cytokines and oxidative stress [19]. Therefore, the temporal dynamics and concentration levels of epinephrine are key factors determining whether it enhances or inhibits memory. Only a moderate, short-lived surge maximizes memory benefits, whereas prolonged activation leads to maladaptation and cognitive impairment, see Figure 1.

## 3. Timing Determines Efficacy: The “Window Effect” of Post-Learning Epinephrine Surge

### 3.1. The Critical Time Window for Memory Consolidation

Memory consolidation is a dynamic neurobiological process that transforms brief, fragile new memories into stable, long-lasting long-term memories. This process is not instantaneous but requires hours or even days, during which memory representations are highly plastic, forming a critical “time window” [20]. Interventions during this window can maximize their impact on the final strength of the memory. From a neural mechanism perspective, memory consolidation involves complex interactions between the hippocampus and the cerebral cortex: the hippocampus is initially responsible for rapidly binding memory elements, after which, through a series of molecular and cellular events, the memory gradually becomes stored in distributed cortical networks independent of the hippocampus [2]. The adrenergic system plays a key regulatory role in this process. Studies show that increasing epinephrine levels immediately after learning significantly enhances memory retention, whereas delaying administration by several hours greatly reduces or completely abolishes this effect [13]. This time-dependent effect confirms the existence of a limited sensitive period for memory consolidation, during which epinephrine acts as a powerful modulator, enhancing the stability of memory traces by activating β-adrenergic receptors in the hippocampus and amygdala [16]. The discovery of this time window provides a solid theoretical basis for post-learning intervention strategies, explaining why precise timing is crucial for memory enhancement effects [21].

### 3.2. The “Increment Effect” of the Epinephrine Surge

The key factor determining the memory enhancement effect is not the absolute concentration of epinephrine, but its relative change, i.e., the “increment effect” [22]. This core concept emphasizes that a significant post-learning rise in epinephrine levels, creating a strong contrast against the lower baseline level during learning, is the key signal for effectively triggering memory-strengthening mechanisms. The underlying neural mechanism is that a moderate baseline epinephrine level primarily maintains a neural state conducive to information encoding, whereas the sharp peak after learning can more potently activate β-adrenergic receptors in the hippocampus and amygdala, triggering stronger cAMP/PKA signaling pathway activity. This, in turn, leads to large-scale regulation of immediate-early gene expression and protein synthesis, molecular events critical for stabilizing synaptic changes and strengthening long-term potentiation [23]. Research by Cahill and Alkire provides direct evidence for this; they found that the memory-enhancing effect of post-learning administration of an adrenergic agonist was most pronounced in a relatively low-arousal encoding context, as this created the greatest “neurochemical contrast” [24]. This increment mechanism has important evolutionary significance: it ensures the organism prioritizes the consolidation of memories related to important events (possibly signaling threat or reward) encountered after a period of calm, thereby optimizing future survival decisions. If epinephrine levels remain consistently high both during and after learning, the relative change is small, failing to provide an effective strengthening signal, which explains why chronic stress impairs memory function.

### 3.3. Avoiding Interference During the Learning Process

A key advantage of strictly timing the epinephrine surge to occur after learning, rather than during the learning process itself, is avoiding the potential interference that high epinephrine levels can have on the information encoding phase. Memory encoding requires high concentration and effective allocation of cognitive resources. Excessively high epinephrine levels during learning may induce a state of over-arousal, characterized by increased anxiety and a shift in attention away from the learning task itself towards internal or external stressors, thereby reducing the efficiency and quality of information encoding [25]. The prefrontal cortex, responsible for higher executive functions like working memory and attentional control, is highly sensitive to changes in epinephrine levels. According to the Yerkes–Dodson law, excessively high arousal levels impair prefrontal cortex function, causing cognitive performance to fall from the peak of the “inverted U-shaped” curve to its descending limb [17]. Studies show that administering β-adrenergic receptor blockers before or during learning typically does not affect memory encoding, but administering them after learning blocks memory consolidation [26]. This conversely demonstrates that high adrenergic activity during the encoding phase is not necessary and may even be detrimental. The post-learning surge strategy achieves functional separation: allowing encoding to occur in a calm, focused neurochemical environment, followed by an epinephrine “shock” to strengthen the already encoded information, thereby maximizing the benefit for the entire learning and memory process. This precise temporal arrangement ensures epinephrine exerts its most appropriate role at each stage of memory formation.

## 4. The Dual Advantage of Exercise: Efficient Epinephrine Surge and Synergistic Neural Benefits

### 4.1. Rapidity and Safety of Exercise-Induced Epinephrine Release

**Figure 2:**
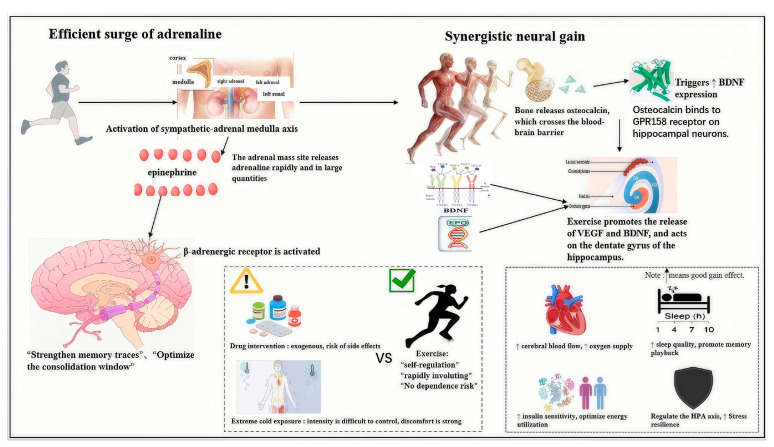
Double advantages of exercise in enhancing learning and memory: acute epinephrine surge and multi-channel synergistic neural gain. This figure shows how exercise, as an ideal intervention strategy, enhances the memory effect after learning through the’ dual advantage’ mechanism. (left) Advantage one: safe, rapid, and controllable induction of acute epinephrine release, accurate matching of memory consolidation time window. (Right) Advantage 2: Beyond epinephrine, it has broad and lasting benefits to the brain through multiple pathways, such as osteocalcin signaling, promoting neurogenesis, and system optimization. Original integrative diagram illustrating the dual advantages of exercise in enhancing memory consolidation.

Exercise, as a natural, non-pharmacological physiological stressor, can safely, controllably, and efficiently activate the sympathetic nervous–adrenal medulla system, prompting rapid epinephrine release, thereby providing an ideal neurochemical environment for post-learning memory consolidation [27]. Compared to pharmacological interventions or extreme cold exposure, exercise-induced epinephrine release offers unique advantages: its intensity can be precisely modulated by exercise type, duration, and intensity, and it follows natural physiological feedback mechanisms, avoiding potential side effects or dependency risks associated with exogenous substances [28]. Physiologically, increased muscle contraction and metabolic demands during exercise activate cardiovascular centers, prompting the adrenal medulla to release catecholamines such as epinephrine and noradrenaline via β-adrenergic signaling pathways, typically reaching peak levels within 5–10 min after exercise onset [29]. This release pattern highly coincides with the memory consolidation “time window,” making exercise an ideal intervention. Studies show that moderate-intensity exercise, for example, running or cycling at 60–70% of maximum heart rate, can increase plasma epinephrine concentration by 2–4 times, an amplitude precisely within the optimal range for enhancing memory consolidation, avoiding anxiety and distraction from over-activation while providing sufficient chemical signals to strengthen memory traces [30]. Furthermore, epinephrine levels naturally decline after exercise, meeting the requirement for an “acute surge” rather than “chronic elevation,” avoiding the neurotoxic effects of long-term high epinephrine levels on the hippocampus. Compared to stimulants like caffeine, exercise can also promote subsequent recovery processes, inducing the release of endogenous opioids to produce a sense of pleasure that counteracts discomfort from the stress response, thereby improving intervention adherence and sustainability [31]. Notably, the safety of exercise is also reflected in its self-regulating nature; individuals naturally reduce exercise intensity when feeling discomfort, an intrinsic protective mechanism absent in pharmacological interventions [32]. See Figure 2.

### 4.2. Beyond Epinephrine: Multi-Pathway Synergistic Regulation of the Memory System by Exercise

The memory-enhancing effects of exercise extend far beyond epinephrine release. Its unique value lies in providing comprehensive support for memory formation through multi-system, multi-pathway synergistic actions. Firstly, exercise induces changes in skeletal endocrine function, particularly promoting the secretion of osteocalcin by osteoblasts, a hormone recently discovered to have profound effects on cognitive function [33]. After aerobic exercise, the uncarboxylated form of osteocalcin enters the bloodstream, crosses the blood–brain barrier, and directly binds to GPR158 receptors on hippocampal neurons, enhancing BDNF expression and promoting synaptic plasticity and neuronal survival [34]. Animal experiments show that injecting osteocalcin can mimic the cognitive improvements from exercise, while osteocalcin-deficient mice cannot benefit from exercise, confirming osteocalcin’s key mediating role in the exercise-cognition link [35]. Secondly, exercise, particularly regular aerobic exercise, significantly promotes neurogenesis in the hippocampal dentate gyrus, a process crucial for certain types of learning and memory [36]. Exercise promotes the release of Vascular Endothelial Growth Factor through increased blood flow shear stress and induces hippocampal BDNF expression, creating a favorable microenvironment for neural stem cell proliferation and differentiation [37]. Research finds that moderate-intensity aerobic exercise performed 3–5 times per week for 30–45 min can increase hippocampal volume by 1–2%, equivalent to reversing 1–2 years of age-related atrophy [38]. Thirdly, exercise supports cognitive function systemically through various indirect mechanisms: improving cardiovascular health and cerebral blood flow perfusion, ensuring energy and oxygen supply; modulating hypothalamic–pituitary–adrenal axis function, enhancing stress resilience; promoting slow-wave sleep activity, strengthening memory replay and consolidation; increasing insulin sensitivity and cerebral glucose metabolism, optimizing brain energy utilization efficiency [39]. This multi-target action makes exercise a unique “broad-spectrum” cognitive enhancement strategy, whose effects far exceed those of a mere epinephrine surge.

### 4.3. Optimizing Exercise Intervention Strategies: Type, Dose, and Timing

Based on current evidence, optimizing exercise intervention strategies requires precise consideration of three key dimensions: exercise type, dose, and timing, to maximize its promoting effect on memory consolidation. Regarding exercise type, moderate-intensity continuous aerobic exercise, such as brisk walking, jogging, swimming, and cycling, shows the most consistent beneficial effects, as it effectively elevates epinephrine levels, promotes osteocalcin release, and induces neurogenesis simultaneously [40]. Although high-intensity interval training can also significantly elevate epinephrine, its promoting effects on BDNF and neurogenesis are relatively weaker, and it may cause excessive fatigue affecting subsequent cognitive function due to its high intensity [41]. While resistance training benefits musculoskeletal health, its effects on improving hippocampal function and memory are less pronounced than aerobic exercise, suggesting it should be used as an adjunct rather than the primary intervention method [42]. Regarding dose, based on meta-analysis evidence, performing moderate-intensity aerobic exercise 3–5 times per week, for 20–45 min per session, which accumulates 150–200 min weekly, is recommended, as this dose produces significant and sustainable cognitive benefits [43]. Exercise intensity should be controlled at 60–75% of maximum heart rate or at a subjective level of “somewhat hard but still able to talk,” a range that optimizes epinephrine release without causing excessive stress responses [44]. Most crucially, timing selection exercise should be performed immediately after learning concludes, ideally within 30 min to precisely match the sensitive time window for memory consolidation [45]. Studies find that subjects performing 30 min of moderate-intensity aerobic exercise immediately after learning had a 10–15% higher memory retention rate after 48 h compared to delayed exercise groups or sedentary control groups [46]. This timing-specific effect is closely related to the epinephrine “increment effect” and the temporal coupling between exercise-induced molecular events, such as BDNF and osteocalcin release, and the memory consolidation process. Therefore, an optimized exercise intervention plan should be an integrated strategy based on aerobic exercise type, moderate-intensity dose, and immediate post-learning implementation.

## 5. From Theory to Practice: Application Prospects and Potential Challenges

### 5.1. Application Potential in the Fields of Education and Vocational Training

Integrating the “post-learning exercise” intervention strategy into education and vocational training systems demonstrates broad application prospects as a low-cost, high-efficiency cognitive enhancement tool [47]. This neuroscience-evidence-based method can significantly optimize the efficiency of knowledge acquisition and skill mastery, particularly in learning scenarios requiring substantial memorization and skill consolidation [48]. In the educational field, brief “exercise micro-breaks” can be embedded into curriculum design—for instance, scheduling 10–15 min of moderate-intensity physical activity such as brisk walking, aerobics, or jumping rope immediately after a 45-min main lesson. This not only reinforces recently learned knowledge but also alleviates fatigue from prolonged sitting and improves attention for subsequent learning [49]. Empirical studies show that schools adopting such models see significant improvements in students’ memory retention rates and test scores, with effects being most pronounced in subjects like language learning and factual memorization [50]. This strategy holds significant value in higher education and professional training as well: medical students could participate in directed physical activity after anatomy lectures to enhance memory of complex structures; language learners could use moderate exercise after vocabulary study to strengthen memory traces [51]. Vocational skill training, especially for procedural skills requiring motor memory, such as instrument operation and surgical skills, can also benefit from post-learning exercise interventions, as exercise enhances not only declarative memory but also the consolidation of procedural memory [52]. From an implementation perspective, the cost-effectiveness ratio of this strategy is highly attractive: it requires no expensive equipment or medication, only reasonable scheduling of time and space, and the physical health benefits from exercise can further reduce healthcare costs [53]. With the popularization of distance learning and blended education, corresponding guidance programs can be developed to encourage learners to engage in home-based exercise after online courses, thereby compensating for the lack of physical activity in virtual learning environments [54]. See Table 1.

### 5.2. Challenges and Future Directions for Personalized Programs

Although the overall effect of exercise on memory enhancement is confirmed, significant individual differences exist in the response to exercise interventions, making the development of personalized programs a key challenge and future direction for achieving optimal outcomes. This individual variability stems from multiple factors: regarding age factors, adolescents and older adults respond differently to exercise type and intensity [55], with adolescents potentially benefiting more from higher-intensity exercise, while older adults require consideration of musculoskeletal safety and cardiovascular limitations [56]; gender differences manifest as variations in physiological response and cognitive benefits to exercise across different phases of the menstrual cycle in females [57]; baseline health status, such as body mass index, cardiovascular fitness level, and the presence of metabolic diseases, influences individual responses to exercise; genetic factors, like the BDNF gene Val66Met polymorphism, have been shown to affect the degree of exercise-induced neuro-plasticity and cognitive improvement [58]. Facing these challenges, future research needs to explore precise exercise “prescription” plans, tailoring the most suitable exercise parameters such as type, intensity, duration, and frequency based on individual physiological characteristics, genotype, and preferences [59]. Digital health technologies provide powerful tools. For personalization: monitoring real-time physiological metrics, for example, heart rate variability, exercise intensity via wearable devices, combined with machine learning algorithms, can enable dynamic adjustment of exercise prescriptions to adapt to an individual’s immediate state and long-term adaptation changes [60]. Furthermore, considering individual chronotypes, including morning/evening types and psychological characteristics such as exercise motivation and self-efficacy, will also help improve intervention acceptance and effectiveness [61]. Future research requires large-sample randomized controlled trials using factorial designs to explore the optimal exercise parameters for different populations and to build predictive models identifying individual characteristics most likely to benefit from specific exercise regimens [62].

### 5.3. Long-Term Adherence and Behavior Change

Translating “post-learning exercise” from a short-term intervention into a long-term, sustainable lifestyle change faces significant behavioral adherence challenges, requiring the integrated application of behavioral science theories and technological innovation to 341 facilitate habit formation and maintenance. Low exercise adherence is a global public health problem, with studies showing over 50% of individuals discontinue an exercise plan within 6 months of starting [63]. Barriers to long-term adherence include time constraints, lack of motivation, environmental obstacles, and insufficient self-regulatory capacity [64]. To address these challenges, multi-level strategies can be designed based on behavior change theories such as, COM-B model, Self-Determination Theory: at the individual level, enhancing self-efficacy through goal setting, self-monitoring, and immediate feedback [65]; at the social level, creating social identity and accountability using peer support, group exercises, and social media sharing [66]; at the environmental level, optimizing the design of learning and workspaces to provide convenient exercise facilities and prompts such as, stair markings, standing desks [67]. Technological innovations offer novel solutions for improving adherence: smartphone applications can provide personalized reminders and adaptive exercise recommendations; wearable devices can enhance exercise enjoyment through gamification elements such as achievement badges, progress bars [68]; virtual reality technology can create immersive exercise experiences, making indoor exercise more enjoyable and varied. Additionally, integrating exercise into daily routines, such as “active commuting” and “lunchtime walks,” rather than treating it as an additional burden, helps improve long-term sustainability [69]. Importantly, exercise programs should offer sufficient flexibility and choice, allowing individuals to adjust intensity based on their daily state, voiding an “all-or-nothing” mindset, which facilitates easier restarting after inevitable interruptions [70,71]. Future research needs to explore how to seamlessly integrate exercise interventions into the daily lives of different populations and assess the long-term effects and their impact on cognitive health. See Table 2.

## 6. Conclusions and Outlook

This article elucidates the neural mechanisms through which exercise promotes post-learning memory consolidation by inducing acute epinephrine release. It underscores the critical role of moderately elevated acute epinephrine in enhancing synaptic plasticity and LTP via the activation of hippocampal β-adrenergic receptors, while also highlighting the significant temporal and dose dependency of its effects. As a safe physiological intervention, evidence suggests that exercise can precisely elicit a post-learning epinephrine surge and exerts broad cognitive benefits through synergistic actions involving multiple pathways, such as the osteocalcin signaling pathway, neurogenesis, and systemic metabolic optimization. Furthermore, this article proposes optimized strategies based on exercise type, intensity, and timing, emphasizing the particular importance of immediate post-learning, moderate intensity aerobic activity, and demonstrates its application potential in scenarios such as education and training.

Future research should focus on developing individualized “exercise prescriptions” that account for factors like genetics, age, and health status. This requires precisely quantifying dose–response relationships through large-scale experiments and computational modeling. There is also a need to deeply explore the synergistic effects of exercise combined with other interventions and to leverage technologies like wearable devices and virtual reality to enhance intervention adherence and sustainability. Through interdisciplinary collaboration and translational research, this strategy holds promise for providing scientific, effective, and scalable practical solutions for promoting cognitive health.

## 7. Limitations

While this review has systematically synthesized evidence supporting the role of exercise-induced epinephrine in enhancing post-learning memory consolidation, several inherent limitations should be acknowledged. The primary limitation stems from the narrative synthesis approach adopted, which, while comprehensive, inherently lacks the quantitative rigor of a meta-analysis. This limitation is largely attributable to the substantial heterogeneity observed across the existing literature, including variations in study designs, exercise protocols (encompassing type, intensity, and duration), and memory assessment tools. Consequently, the conclusions drawn regarding key parameters—such as the optimal post-learning exercise “time window” and the comparative efficacy of modalities like MICT versus HIIT are necessarily qualitative and lack precise quantitative effect sizes. Furthermore, this review is constrained by the current scope and depth of the primary literature. Critical discussions on individual differences—such as those related to age, sex, and genetic background—remain largely descriptive due to a paucity of controlled studies designed to directly compare and disentangle these interacting factors. Finally, our synthesis of the underlying neurobiological mechanisms, though outlining key pathways like BDNF and osteocalcin, is limited by the scarcity of integrated human studies that concurrently measure peripheral epinephrine release, central molecular changes, and behavioral memory outcomes, thereby leaving the precise causal pathways partially inferred.

## Figures and Tables

**Table 1:** Application Potential and Implementation Suggestions of the “Post-Learning Exercise” Strategy in Different Fields.

Application Field	Specific Application Scenarios	Implementation Suggestions	Expected Benefits
**Basic Education**	After classroom teaching	Schedule 10–15 min of moderate-intensity activity, such as brisk walking, aerobics, jumping rope, after main lessons	Reinforces knowledge and memory, alleviates sedentary fatigue, improves subsequent attention, and significantly enhances memory retention rates and test scores
**Higher Education and Professional Training**	After language learning, skill training, and lectures	Conduct directed physical activity, such as 20–30 min of jogging or ball sports, after lectures or training sessions	Enhances memory of complex knowledge structures; promotes consolidation of procedural motor skills, such as instrument operation
**Remote and Blended Learning**	After online courses	Develop home-based exercise guidance programs, such as follow-along videos and app reminders	Compensates for the lack of physical activity in virtual learning environments and extends cognitive benefits to online learning scenarios
**Universal Value**	Curriculum and health policy design	Integrate “exercise micro-breaks” into timetables and teaching plans	Low cost, high benefit. Requires no expensive equipment/medication, combines dual advantages of enhancing cognition and promoting physical health, and reduces medical expenditure

**Table 2:** Main Challenges and Response Directions for Implementing the “Post-Learning Exercise” Strategy.

Challenge Category	Specific Manifestations and Root Causes	Individualized and Precise Coping Strategies	Behavioral Change and Technical Support Strategy
**Individual Response Differences**	Effectiveness is influenced by age, gender, baseline health status, and genetic factors such as brain-derived neurotrophic factor gene polymorphism; “one-size-fits-all” plans have limited effect.	Develop precise exercise “prescriptions”: customize exercise parameters (type, intensity, duration) based on physiological characteristics, genotype, and preferences. Use digital health technology such as wearables + machine learning for dynamic adjustment.	Consider psychological characteristics (motivation, self-efficacy) and temporal preferences (morning/evening type) to improve plan acceptance.
**Insufficient Long-** **Term Adherence**	>50% of individuals quit an exercise plan within 6 months. Barriers include time constraints, lack of motivation, environmental obstacles, and insufficient self-regulatory capacity.	Offer flexible plans, allow intensity adjustment based on state, avoid “all-or-nothing” mindset, and facilitate restarting after interruptions.	Multi-level Behavioral Interventions: (1) Individual level: Goal setting, self-monitoring, and immediate feedback. (2) Social level: Peer support, group exercise, and social sharing. (3) Environmental level: Optimize venue design with features like standing desks and prominent stairs.
**Technology Integration and Sustainability**	Integrating interventions seamlessly and enjoyably into daily life is a major challenge.	/	Technological Innovation and Gamification: (1) Mobile apps: Personalized reminders and recommendations. (2) Wearable devices: Gamification elements, such as achievement badges and progress bars. (3) Virtual reality (VR): Create immersive, enjoyable exercise experiences. (4) Habit fusion: Combine exercise with daily routines, such as active commuting or lunch walks. Do not treat exercise as an extra burden.

## Data Availability

No new data were created or analyzed in this study. Data sharing is not applicable to this article.
